# Symptomatic Pneumocephalus Associated with Lumbar Dural Tear and Reverse Trendelenburg Positioning: A Case Report and Review of the Literature

**DOI:** 10.1155/2013/792168

**Published:** 2013-12-22

**Authors:** Stephen M. Pirris, Eric W. Nottmeier

**Affiliations:** ^1^Department of Neurosurgery, Mayo Clinic, 4500 San Pablo Road, Jacksonville, FL 32224, USA; ^2^St. Vincent's Brain and Spine Institute, 3 Shircliff Way, Jacksonville, FL 32204, USA

## Abstract

Symptomatic pneumocephalus is a rare complication of degenerative lumbar spine surgery. This is a case report of a patient who developed transient diplopia associated with pneumocephalus following lumbar spine surgery complicated by a dural tear. The diplopia improved as the pneumocephalus resolved. Factors involved in the development of pneumocephalus include an unintended durotomy and intraoperative reverse Trendelenburg positioning that was utilized to decrease the risk of postoperative vision loss. When encountering cerebrospinal fluid (CSF) leakage intraoperatively, spine surgeons should level the operating table until closure of the dural defect to prevent potential complications associated with pneumocephalus. If postoperative patients complain of severe headaches or display a focal cranial neurologic deficit, then a computed tomography (CT) scan of the brain should be ordered and evaluated. Consulting neurologists should be aware of the circumstances surrounding this rare complication.

## 1. Introduction

Symptomatic pneumocephalus is a rare complication of degenerative lumbar spine surgery. This is a case report of a patient who developed transient diplopia associated with pneumocephalus following lumbar spine surgery, which then improved as the pneumocephalus resolved. Factors involved in the development of pneumocephalus included an unintended durotomy and reverse Trendelenburg positioning that was utilized to decrease the risk of postoperative vision loss. As spine surgeons increasingly position patients in this fashion, the risk of pneumocephalus associated with dural tears will correspondingly increase. Therefore, the recommendation is to level the operating table when a dural tear is encountered and to raise the head of bed only after satisfactorily closing the dural defect. Consulting neurologists for patients in the postoperative period may benefit from being aware of intraoperative reverse Trendelenburg positioning possibly contributing to the risk of pneumocephalus.

## 2. Case Presentation

**Figure 1:**
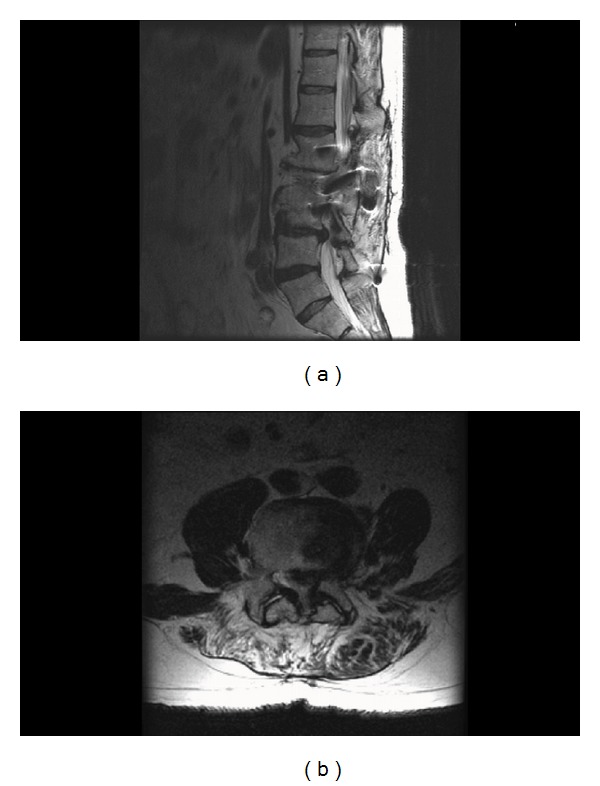
Sagittal (a) and axial (b) T2 weighted MRI scan of the lumbar spine displaying segment degeneration adjacent to a previous instrumented fusion with disc herniation as well as facet and ligamentum flavum hypertrophy.

A 65-year-old male presented with a surgical history of an L2–4 posterior instrumented arthrodesis performed at a local hospital. Previous history included a vitreous humor hemorrhage in the left eye, resulting in slightly decreased vision in that eye. The patient is a former professional golfer. He was able to return to playing golf approximately six months after his previous surgery. Over the past year, he had a significant decline in his ability to ambulate due to recurrent low back pain radiating down both lower extremities, which was consistent with lumbar pseudoclaudication. He had retired from golf and had not been able to play casually for six months due to the pain. Imaging revealed adjacent segment degeneration with disc herniation as well as facet and ligamentum flavum hypertrophy (Figure 1). The patient failed conservative treatments with formal physical therapy and multiple injections. The decision was made to undergo a reexploration for removal of instrumentation, L4-5 discectomy, and extension of fusion to L5 with pedicle screws and interbody cage placement.

During the surgery, the patient was positioned prone on the Jackson Table (Mizuho OSI, Union City, CA). For prevention of the rare complication of blindness during prolonged spine surgery [1], we routinely employ a Mayfield skull clamp (Integra Life Sciences, Plainsboro, NJ) and keep at least 5° of reverse Trendelenburg positioning on patients undergoing instrumented lumbar fusion. An unintended durotomy was made while dissecting scar tissue off the posterolateral dura. After dissecting more scar tissue in order to expose the defect, it was closed primarily with 6-0 prolene suture. Valsalva maneuver did not express any cerebrospinal fluid (CSF), and the remainder of the surgery was completed without further complications.

**Figure 2:**
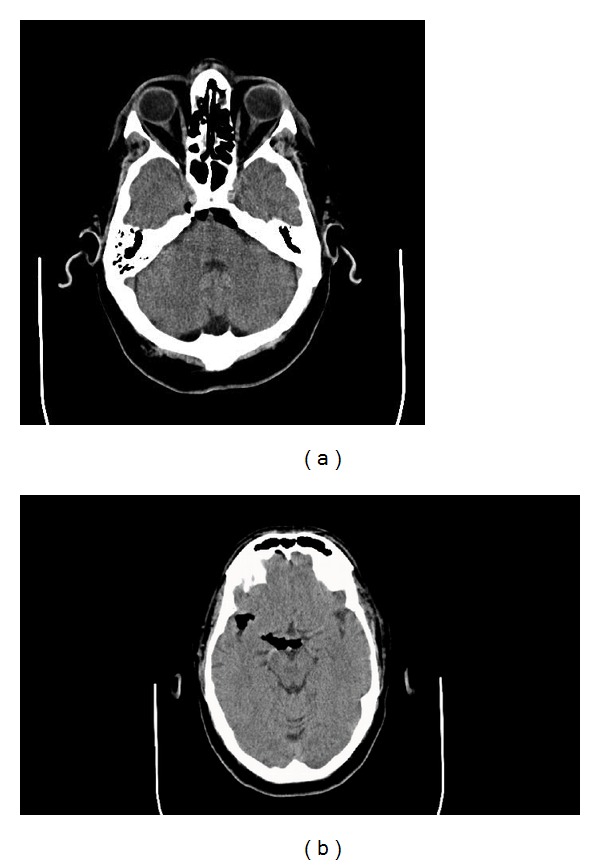
Noncontrast CT scan of the brain displaying significant pneumocephalus exerting slight mass effect on the pons.

**Figure 3:**
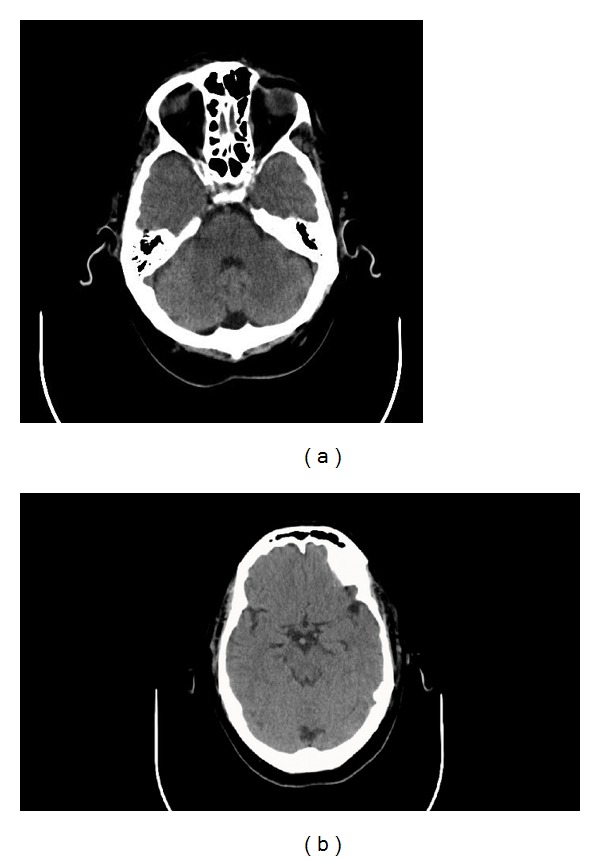
CT head scan on postoperative day 2 showed resolution of the pneumocephalus.

In the postanesthesia recovery room, the patient complained of diplopia and displayed a left 6th nerve palsy on exam. A computed tomography (CT) head scan without contrast was obtained and revealed significant pneumocephalus exerting a slight mass effect on the pons (Figure 2). The patient was transferred to the surgical intensive care unit for observation and treatment with 100% oxygen per institutional pneumocephalus protocol. His diplopia resolved overnight, and a repeat CT head scan on postoperative day 2 showed resolution of the pneumocephalus (Figure 3). The patient denied any headache or other symptoms of pneumocephalus. He ambulated well after 24 hours of bed rest and was discharged home on postoperative day 4 with no evidence of CSF leak. There were no long-term sequelae of the dural tear or pneumocephalus noted at his 6-week and 3-month follow-up appointments. He returned to playing golf recreationally.

## 3. Discussion

Dural tears encountered during degenerative lumbar spine surgery have been reported to occur in 1.8% to 17.4% of cases [2, 3]. The largest currently known study to determine the incidence of dural tears was published by Khan et al. in 2006 [2]. In their study, the incidence of dural tears in primary lumbar surgeries was found to be 7.6%, and their incidence during revision lumbar surgeries was 15.9% [2].

There have been multiple reports in the literature of symptomatic pneumocephalus occurring after lumbar puncture, lumbar epidural steroid injection [4], and continuous lumbar drainage [5, 6], but the occurrence after unintended durotomy is less frequently reported. In a 1967 review of 295 previously reported cases of pneumocephalus, 11 (3.7%) were related to surgical intervention, but none of these were spinal operations [7]. This case collection did include three cases related to lumbar puncture. There have been published case reports of patients who developed pneumocephalus and meningitis after unrecognized dural violation [8, 9].

Intracranial hypotension has been reported as a cause for chronic headaches in patients who suffered CSF leaks during spinal surgery [10–12]. Inamasu and Guiot identified nine published cases of intracranial hypotension that was diagnosed after spinal surgery, including three that developed subdural hematomas after lumbar discectomy [12].

Symptomatic pneumorachis has been reported previously in a patient who woke up intraoperatively during an unintended durotomy repair [13]. In this patient, aspiration of air through the dural defect during the accidental awakening had been postulated as the cause of the gaseous intraspinal collection.

There have been several reports of remote cerebellar hemorrhage associated with dural tears during spine surgery [11]. The cause of the hemorrhages is presumed to be due to intracranial hypotension caused by the loss of CSF through the dural tear.

Theories on the development of pneumocephalus include (1) a ball valve mechanism where air enters the CSF space through a dural rent but does not escape and (2) an inverted bottle mechanism where no further leakage of spinal fluid occurs unless air enters the CSF space and allows the dependent fluid to flow [11]. The inverted bottle theory applies to our patient.

The reason for intraoperative reverse Trendelenburg positioning is to mitigate the risk of perioperative vision loss, which is another rare but devastating complication. Vision loss has been reported in the literature by several case reports and case series [1]. The American Society of Anesthesiologists developed a task force to produce a practice advisory in 2006. They published a report indicating that the main risk factors are associated with vascular factors. They agree that (1) the preoperative presence of anemia, (2) prolonged procedures, (3) substantial blood loss, and (4) prolonged procedures combined with substantial blood loss all increase the risk of perioperative visual loss [1]. There is no evidence that ocular compression causes isolated perioperative anterior ischemic optic neuropathy (ION) or posterior ION. However, direct pressure on the eye should be avoided to prevent central retinal artery occlusion (CRAO). The high-risk patient should be positioned so that the head is at level with or higher than the heart when possible, and the highest-risk patient should be positioned with the neck in neutral position without significant flexion [1]. Other intraoperative measures recommended by this committee include the following: avoidance of hypotension with continuous blood pressure monitoring; avoidance of anemia with frequent assessments of the hemoglobin and hematocrit (no baseline threshold for transfusion is recommended); colloid solutions should be administered with crystalloid solutions for maintenance of intravascular volume; vasopressors should be administered on a case-by-case basis; and consideration should be given to staged spinal procedures in high-risk patients [1].

Treatment of pneumocephalus in our institution involves continuous supplemental 100% oxygen by a nonrebreather mask. The increased oxygen tension in the bloodstream has previously been shown to more rapidly clear the volume of pneumocephalus in case reports and small studies [14, 15]. However, increasing the inspired oxygen beyond 40% may only provide marginal increases in the rate of absorption [14].

## 4. Conclusion

We present this case of a patient with symptomatic pneumocephalus that resolved with conservative management after spine surgery. This paper adds to the literature regarding this infrequent postoperative complication and may enlighten spinal surgeons who utilize the reverse Trendelenburg positioning during surgery and consulting neurologists. Since our experience with this complication, we have asked the anesthesiology team to level the operating bed after dural tears until the dural closure is completed and confirmed with Valsalva maneuver. If a neurologist is consulted on a patient who develops a focal neurologic deficit or complains of headaches after spine surgery that are unresponsive to flat bed rest, hydration, and caffeine, then a CT head scan is indicated to look for the presence of pneumocephalus. If pneumocephalus is noted on CT, then the neurologist may ask the surgeon or anesthesiologist if reverse Trendelenburg position was utilized intraoperatively in order to help decipher the etiology.
